# Antioxidative Molecules in Human Milk and Environmental Contaminants

**DOI:** 10.3390/antiox10040550

**Published:** 2021-04-01

**Authors:** Stefano Lorenzetti, Torsten Plösch, Inga C. Teller

**Affiliations:** 1 Department of Food Safety, Nutrition and Veterinary Public Health, Istituto Superiore di Sanità (ISS), 00161 Rome, Italy; stefano.lorenzetti@iss.it; 2 Perinatal Neurobiology, Department of Human Medicine, School of Medicine and Health Sciences, Carl von Ossietzky University Oldenburg, 26129 Oldenburg, Germany; torsten.ploesch@uol.de; 3 Department of Obstetrics and Gynaecology, University Medical Center Groningen, University of Groningen, 9713 GZ Groningen, The Netherlands; 4 Institute NaturScience, 28199 Bremen, Germany

**Keywords:** breastfeeding, milk composition, MFGM, trace elements, lactoferrin, melatonin, xenobiotics, man-made chemicals, endocrine disruptors, epigenetic modulators

## Abstract

Breastfeeding provides overall beneficial health to the mother-child dyad and is universally recognized as the preferred feeding mode for infants up to 6-months and beyond. Human milk provides immuno-protection and supplies nutrients and bioactive compounds whose concentrations vary with lactation stage. Environmental and dietary factors potentially lead to excessive chemical exposure in critical windows of development such as neonatal life, including lactation. This review discusses current knowledge on these environmental and dietary contaminants and summarizes the known effects of these chemicals in human milk, taking into account the protective presence of antioxidative molecules. Particular attention is given to short- and long-term effects of these contaminants, considering their role as endocrine disruptors and potential epigenetic modulators. Finally, we identify knowledge gaps and indicate potential future research directions.

## 1. Introduction

Human milk is the first option of infant feeding. The World Health Organization (WHO) recommends a breastfeeding duration up to two years with the first six months being exclusive breastfeeding [1]. This recommendation is supported by medical associations such as the American Academy of Pediatrics (AAP) and the European Society for Paediatric Gastroenterology, Hepatology and Nutrition (ESPGHAN) [2,3]. On the other hand, pollutants are known to be present in human milk.

In this narrative review for researchers, medical professionals and policy makers, we take a close look at human milk composition (see Section 2), specifically its bioactive molecules with antioxidative properties (Section 4). Because many environmental and dietary contaminants seem to be associated with the milk fat fraction, we report on milk fat synthesis, the unique characteristics of its architecture (Section 2) and its digestion which affects the access to these fat constructs (Section 3). Environmental and dietary contaminants identified in milk, their potential health impact and interactions with antioxidants and/or signaling pathways are considered (Section 5) before we discuss food safety aspects and monitoring programs during lactation (Section 6). Breastfeeding recommendations (Section 7) in light of these contaminants, including practical suggestions for those concerned, are also included. Finally, an overview of research gaps and future perspectives (Section 8) to support a better understanding of mother’s milk and actions taken to safeguard its quality is provided.

## 2. Human Milk Composition

Human milk composition is exceptionally variable: It varies with environmental factors like climate and season, maternal ethnicity, genetics, health, nutritional status, diet choices, mammary gland development, lactational stage and duration, time of day, time within a feeding session, the infant’s sex and health and is in large modulated to reflect each infant’s individual need [4,5,6]. As consequence, a standardized reference of its composition does not exist.

Furthermore, available data are affected by diversity in milk sampling protocols (lactation stage, time of day, pooling of samples), storage conditions, analytical choices and much more. Some of these variations are illustrated in Table 1.

### 2.1. Milk Fractions

Many nutrients and bioactive compounds enter milk from maternal circulation via receptor-mediated transport. Others are endogenously synthesized by lactocytes, the mammary epithelial cells lining the alveoli, and a subset by the multitude of cell types present freely in milk [6,43,44]. Uptake from the circulation is stimulated by lactational hormones. Glucose transporter expression and activity increases and a multitude of genes are activated that play a role in lactose synthesis, glycolysis, fatty acid transport, de novo lipogenesis, triacylglycerol and protein synthesis or amino acid transport to make this process most efficient [45].

Nutrients or bioactive molecules are roughly distributed over three milk fractions: first, the colloidal dispersion fraction with calcium-rich casein-mineral micelles. Second, the aqueous fraction with small molecules and whey proteins [46,47]. In this second fraction, we find cells and the plethora of water-soluble enzymes (e.g., in Table 1), proteins (e.g., lactoferrin, Section 4.4), peptides, hormones (e.g., melatonin, Section 4.5), water-soluble vitamins (e.g., ascorbic acid), oligosaccharides and immunoglobulins that together contribute to the infection-protective properties of human milk [38,48,49]. The third fraction is milk fat. The fat fraction is of interest in the context of this paper because environmental and dietary contaminants seem to accumulate here: hence, to express lipophilicity of the environmental and dietary contaminants of interest, we reported their octanol-water partitioning ratio in terms of LogP values (Section 5).

### 2.2. Milk Fat Globules

The milk fraction is formed by milk fat globules (MFG). These are complex structures with a unique architecture. MFGs consist of a lipid core that is surrounded by a phospholipid membrane (MFGM). The MFGM is unique in that unlike membranes of cell organelles or chylomicrons used to transport intestinal lipids to the liver, it is not a double-layered but a triple-layered membrane. Functional proteins are embedded. The MFGM creates a polarized layer that allows the dispersion of the lipophilic core in water forming the fat-in-water emulsion that is milk [50,51].

### 2.3. The Fat Core

The fat core is predominantly filled with triacylglycerols, mono- and diacylglycerols from maternal circulation or de novo synthesis and also with fat soluble vitamins, non-esterified fatty acids, cholesterol-esters and other non-polar lipophilic molecules from maternal circulation or de novo synthesis of the rER [45,51,52,53,54].

In contrast to plasma, almost all of the vitamin A in the core (retinol, 95%) is present as retinyl esters, which means the newborn metabolizes a different chemical form of this vitamin after birth [9]. Small amounts of more than 30 carotenoids, flavonoids, γ-tocopherol and ubiquinol (coenzyme 10) are also part of the core [38,46,47]. The carotenoid concentrations are 10 to 120 times lower than those in plasma. [12]. In contrast to the other fat-soluble vitamins, α-tocopherol is associated with the milk fat globule membrane (MFGM) [38].

The core’s fatty acid content is most variable and although de novo synthesis is accelerated to 5-fold higher activity than in the liver [45], it largely translates from maternal diet and body stores. Through a shift towards industrialized foods the fatty acid profile has undergone a remarkable alternation from omega-3 dominant to omega-6 dominant fatty acids over the last six decades with a particular increase of the ratio for the two essential fatty acids linoleic to α-linolenic acid [55]. An extensive overview of human milk fatty acids and according to lactation stage has been published by Floris et al. [56].

The concentrations of many vitamins respond to maternal dietary changes [57,58]. This counts for the fat-soluble vitamins D, E, A and vitamin A precursors, i.e., carotenoids or flavonoids, but also for the water soluble B-vitamins and ascorbic acid. Supplementation can affect translation into milk of other molecules. For example, although baseline concentration of vitamin A and E in seem to be secured in milk of women with poor plasma vitamin status [59], the supplementation of high vitamin A doses reduces α-tocopherol levels in colostrum [60]. The suppression of vitamin E through supplementation of retinol are known from studies in cows [61].

## 3. Digestion of Milk Globules

MFG- and lipid digestion have been extensively discussed by He and colleagues [62] and for details on milk digestion in general we recommend their detailed review. For the purpose of our review we specifically zoom in on milk digestion in early infancy.

When MFG enter the stomach, proteins embedded in the MFGM are partially denatured by the lowered pH and hydrolyzed by gastric pepsin. Interestingly, this process is slow in newborns because the gastric pH is close to neutral and gastric pepsin concentrations are low as well. During the first weeks of life, the gastric pH becomes more and more acidic accompanied by a rise in gastric pepsin production. As a consequence of denaturation and partial hydrolysis proteins and MFG first form large diffuse aggregates and subsequently release the fat globules again. It is then when first steps of lipolysis occur by the action of gastric lipase [62]. Some milk proteins, however, resist the acidic milieu and continue to fulfill their action. A prominent example is lactoferrin (Section 4.4), which under acidic conditions can still transport iron [27].

In adults, bile salts, pancreatic triglyceride lipase and phospholipase A2 significantly contribute to fat digestion. However, their concentrations are very low in the first months after birth. Therefore, fat digestion requires other mechanisms early in life. As consequence, the action of gastric lipase in the stomach is more relevant in infancy than in later life. Moreover, intestinal MFG digestion in infants involves two specific lipases: pancreatic lipase-related protein 2 and bile-salt stimulated lipase, the latter working in the presence of the low bile salt concentrations early in life. Interestingly, bile-salt stimulated lipase can be derived from the infant’s pancreas but is also present in high concentrations in human milk as a product of the mother’s mammary glands. To put it in a simple picture, the mother simultaneously provides both food and cutlery to the infant. Together, these two proteins are predominantly responsible for the lipolysis of MFG during the passage in the alkaline milieu of the small intestine [62]. Ultimately, MFG are split to a variety of differently sized fat micelles, which are known to also contain other hydrophobic molecules including fat-soluble vitamins.

## 4. Human Milk Antioxidants

Human milk contains almost all fat- and water-soluble vitamins at considerable concentrations [7], Table 1. Many of these are known to have antioxidant capacities including vitamins A, C, E and carotenoids. Yet human milk contains many other bioactive compounds besides vitamins that have been attributed with antioxidant capacities (Table 2). This seems to be based on their chemical structure that allows them to directly scavenge reactive oxygen and nitrogen species (ROS and RON, respectively, or “RONS” when taken together [63]. They also modulate redox signaling pathways either by their ability to change activity of the pro-/antioxidant enzymes or that of transcription factors or by acting as transcription factors themselves [63]. The total antioxidant capacity of human milk has been proposed as one of the defense mechanisms by which breastfeeding is protecting infants against infections and diseases [64,65]. However, RONS play a crucial part in many necessary signal transduction pathways so that a balance of RONS concentrations is necessary for a healthy steady-state [63,66].

Assessment of antioxidant efficacy of milk is difficult in vivo because of the high variation of its composition throughout lactation (Table 1), the thousands of molecules that are present or the complex food matrix that human milk comprises with its different fractions [49]. In addition, many of the bioactive molecules are enzymatically cleaved during digestion (such as lactoferrin and α-lactalbumin [31]), extensively metabolized by the liver or kidneys after absorption or fermented by colonic microbiota into (other) bioactive metabolites. Some of these metabolites can turn out to be stable molecules that are accepted players in antioxidant signaling pathways. This concept has been reviewed by Hunyadi [63].

Individual dietary antioxidants per se don’t seem to have enough efficacy to significantly decrease RONS levels in vivo with possible exception of α-tocopherol (vitamin E), which can preserve fatty acid integrity and prevent membrane damage caused by peroxyl radicals (ROO•) [63,64]. However, the combined action of the multiple molecules and their direct interaction or that of their metabolites with redox switches—that is transcription factors regulating enzyme expression—enzyme activation or with redox signaling pathways [63,72] seem to contribute to the redox steady state (or lack thereof).

### 4.1. Signaling Pathways in the Redox Balance

The antioxidant capacities of many vitamins have been extensively reviewed [64,71,73,74,75]. Indirect or non-antioxidant capacities are emerging and these include roles in signal transduction and gene regulation. By capturing ROS, α-tocopherol negatively regulates protein kinase C and phosphoinositide 3-kinase (E.C.2.7.1.137, PI3K)/Stock A strain k thymoma/transforming protein kinase 1 (AKT1) pathways (and others), which are key modulators in signal transduction favorably affected by ROS [73,76]. α-tocopherol can be restored through a cascade involving vitamin C and glutathione illustrated by [77]. Its actions are also mediated through transcription factors that include peroxisome proliferator-activated receptor gamma (PPARγ), nuclear factor kappa B (NFκB) or estrogen receptor beta (ERβ), nuclear factor erythroid 2-related factor 2 (Nrf2) and many others [71,73,78]. Nrf2 binds to the antioxidant response element in the promoter region of many genes and has been termed “a master regulator of antioxidant responses” because it coordinates many defense mechanisms against xenobiotics (Section 5) and oxidative stress [78].

### 4.2. Enzymatic Antioxidant Systems

Enzymatic antioxidant systems in milk include the selenium-dependent glutathione peroxidase (GSHPx, E.C.1.11.1.9), glutathione reductase (GR, E.C.1.6.4.2), glutathione S-transferase (GST, E.C.2.5.1.18), superoxide dismutase (SOD, E.C.1.15.1.1) and catalase (CAT, E.C.1.11.1.6) [22,38]. These systems scavenge radicals and peroxides and prevent their formation [38]. GSHPx, GR and GST as well as enzymes synthesizing GSH are regulated by the transcription factor Nrf2 and thus affected by α-tocopherol concentrations [78] and those of other antioxidants regulating Nrf2 expression and activity. All components for α-tocopherol’s radical scavenging system, e.g., reviewed by [71,73] are present in human milk from GSH and its maintenance enzymes to selenium and vitamin C [79].

### 4.3. Trace Elements

In the dietary context trace elements can be divided into four classes: These are essential trace elements (a minimum is required for health), possibly essential (minimum intake not yet stablished, e.g., chromium, nickel, vanadium), toxic trace elements (those without known health impact yet known toxicities, Section 5.10) and trace elements for which their function remains unknown [20]. Essential trace elements such as selenium, zinc, manganese and iron are often components and cofactors of enzyme systems that scavenge, prevent formation of RONS or restore the system for a repeat process [38,80]. Some of the essential trace elements like selenium depend on maternal status or intake and their supplementation address maternal deficits, only. In contrast, zinc, copper, manganese and iron concentrations in human milk are largely unaffected by maternal status or intake [5].

Selenium is needed especially as selenocysteine. The majority of the 25 known selenoproteins are involved in redox reactions [7]; for example, GSHPx variants require selenocysteine. Dependent on the report, about 20–35%, 9–17% or 4–13% of human milk selenium is associated with the plasma variant of GSHPx, which is also expressed in human milk and can in turn be seen as selenium deliverer to the infant [20,37,38]. Activity of this enzyme is in the range of 25–80 units/L ([20], Table 1) and therefore covers about half of total peroxidase activity in human milk [37]. Presence of selenium also increases uptake of α-tocopherol into milk [38]. This may be explained by the close relation of selenoproteins (GSH and GSHPx) in the recycling of α-tocopherol in the process of ROS detoxification [79]. Dependency of selenium concentrations in human milk on maternal diet explain the high divergence of reported concentrations. These range from 3–84 μg/L with a mean of 16.3 ± 4.7 μg/L (SD) (Table 1, [5,7,20]). However, in contrast to selenium remains the expression of GSHPx independent of lactation stage or parity [37]. Presence of both molecules increases slightly with the fat-rich hindmilk [37] yet GSHPx activity seems to be associated with large molecules in the casein fraction (Table 3, [38]).

Zinc, copper and manganese are cofactors of the respective SOD variants. Human milk provides about 330–390 μg copper, 0.8–4.1 μg zinc and about 4 μg manganese per liter (Table 1, [7]). In milk, copper, zinc and iron are present in the whey and fat fractions alike (Table 3) [81].

Iron is an integral component of many proteins and enzymes most relevant in our context of peroxidase and catalase [27]. Iron concentrations in human milk are low (0.2—0.4 mg/Liter) yet highly bioavailable [7]. Interaction with other molecules is limited for example through chelation by lactoferrin [20] (see Section 4.4).

### 4.4. Lactoferrin

Lactoferrin may be the dominant human milk whey protein but is not specific for this fluid and present in numerous tissues, other biological fluids and especially in specific (secondary) granules of polymorphonuclear neutrophils [28,82]. Anti-microbial, -viral, -carcinogenic, -inflammatory and -oxidant properties have been allocated to lactoferrin with its highest human milk concentration in colostrum, the pre-milk that is expressed in the first postnatal week (Appendix A, Box A1). Since lactoferrin is present in higher molar concentrations than iron, only 3–5% of its iron-binding capacity is used so that its other modes of action significantly contribute to the immune protection capacity of human milk [27,83]. Lactoferrin seems to add to the good bioavailability of iron in human milk that may be mediated in part via lactoferrin transport, which is active under a large pH range, and may also regulate duodenal iron absorption via specific lactoferrin receptors [20,27].

Lactoferrin binds two ferric iron (Fe^3+^) ions or two ions of copper, zinc and manganese reversibly but with lower affinity [27]. This capacity removes metal cations from a potential Fenton reaction and thus interrupts ROS formation. In addition, lactoferrin activates monocytes to bind iron and by interfering in the binding of L-selectin to lipopolysaccharides lactoferrin prevents a neutrophil oxidative burst. These mechanisms protect (membrane) lipids from oxidative damage [27,84,85]. Another proposed antioxidative mechanism is trace element ion-independent and mediated via stimulation of glycolysis. Lactoferrin would act here as acceptor in the electron transport chain. Such stimulation increases concentrations of ATP, which are required for iron gradient maintenance and that of the membrane potential [85]. Lactoferrin supplementation increases total hydrophilic antioxidant capacity as well [84]. Especially when free of iron lactoferrin downregulates the mitogen-activated protein kinase (MAPK) and NFκB signaling pathways involved in inflammation and balances the redox load through upregulation of Nrf2 protein expression and that of GSHPx, GST and heme oxygenase-1 [86,87]. The Nrf2 upregulation is likely indirect and mediated via expression of erythropoietin through the redox-sensitive transcription factors hypoxia-inducible factor (HIF) 1α and 2α, which are stabilized by lactoferrin [87]. Erythropoietin belongs to the cytokine I superfamily, has been described as a “multifunctional trophic factor exerting control over the homeostasis of the entire organism” including the nervous system and plays a crucial role in response to hypoxia [87].

### 4.5. Melatonin

Melatonin is a well-known circadian hormone and expressed along the circadian rhythm in significant concentrations in human milk (Table 1) [34,88]. Melatonin is regulating metabolism and acting via nuclear receptors that are constituting the circadian clock [88,89]. By regulating the circadian rhythm and thereby reducing chronodisruption, fewer oxidative reagents are generated [69]. Its antioxidant functions have been reviewed extensively [69,90]. Because its first, second and third tier metabolites retain their scavenger function, melatonin induces an antioxidant cascade that quenches ten radical products, which makes it more efficient than glutathione [69,90].

Besides interaction with radicals (•OH, ROO•), RONS and other oxygen-containing molecules (H_2_O_2_, ^1^O_2_), it chelates heavy metals such as copper, lead, ferrous (Fe^2+^) and Fe^3+^ iron—the latter with an activity that may be higher than that of α-tocopherol and thus prevent potential oxidative damage [69,90]. Melatonin is not only synthesized in the pineal gland but also in mitochondria of many tissues, where it protects ATP synthesis, mitochondrial function and integrity. It upregulates glutathione synthesis and that of glutathione disulfide reductase (GSR, E.C.1.8.1.7) and catalase and suppresses xanthine oxidase (XDH, E.C.1.17.3.2.) and nitric oxide synthase (NOS, E.C.1.14.13.39) [69,90,91]. This activation of antioxidant enzymes is at least partially mediated by Nrf2. The melatonin-Nrf2 axis has been recently described in detail by Ahmadi and Ashrafizadeh [92]: melatonin can directly interact with Nrf2 leading to activation of SOD, CAT and GSH hence counteracting oxidative stress. On the other hand, it can indirectly activate Nrf2, for example via protein kinase C (in the pancreas) or PI3K and protein kinase B (in the liver) [92].

## 5. Environmental and Dietary Contaminants

Besides the nutrients, vitamins and antioxidants introduced in chapters 1–3, it is not surprising that milk also contains less desired molecules, e.g., xenobiotics. By definition, xenobiotics are synthetic chemicals that can be found in any organism yet are not produced by the organism itself. Differently from endogenously produced molecules, environmental xenobiotics in air, water and soil are taken up by organisms through ingestion, inhalation or dermal absorption and enter the human food chain. Generally, the population is chronically exposed to very low concentrations of xenobiotics due to the worldwide release of chemicals from consumer products. The latter include personal care products and cosmetics (e.g., parabens), disinfectants (e.g., triclosan), plant protection products (i.e., herbicides, insecticide, biocides), pharmaceutical drugs, plastics (e.g., phthalates, bisphenols) as well as many others used in industrial processes and as components of other products (e.g., paints, electrical devices, cars and so on) [93].

Humans are mainly exposed to xenobiotics through the food chain [94,95,96,97,98,99,100,101,102]. Subsequently, these xenobiotics are described as environmental and dietary contaminants [103,104]. In addition, some of them such as bisphenol A (BPA) and several phthalates have been recognized as “endocrine disruptors” (EDs). An ED is “an exogenous substance or mixture that alters function(s) of the endocrine system and consequently causes adverse health effects in an intact organism, or its progeny, or (sub) populations” (Appendix B, Box A2) [105].

### 5.1. Critical Windows of Development—Developmental Origins of Health and Disease Concept

Modification of diet and different dietary intake during the perinatal period can lead to epigenetic effects—the molecular phenomena acting on gene expression without nuclear DNA alteration (mutations). From a nutritional perspective, the existence of epigenetic changes mediating the interaction of gene and environment in humans have been supported by epidemiological studies considering both fetal undernutrition (as in the Dutch famine birth cohort in the 2nd World War) and over-nutrition (as a consequence of maternal obesity or diabetes) [106]. The possibility to compensate epigenetic effects of an adverse intrauterine environment by dietary interventions has been suggested but conclusive studies on the reversibility of fetal programming in humans are missing [107].

Similarly, exposure to environmental and dietary contaminants can lead to epigenetic effects, even with transgenerational consequences. Transgenerational effects from prenatal exposure to contaminants have been shown at first in experimental animal models exposed to the pesticide vinclozolin, a progenitor of EDs acting as epigenetic regulator. Vinclozolin was able to alter DNA methylation signatures at the third generation from the triggering exposure without any signs in the previous generations [108]. Further studies led to the consideration of vinclozolin as an example of a xenobiotic leading to gene- and life stage-specific effects on metabolic programming [109].

Exposure to environmental and dietary contaminants from conception to completed sexual maturation are critical because such periods of development have been described as sensitive programming windows by the Developmental Origins of Health and Disease (DOHaD) concept. This concept describes how interference during programming windows can cause chronic or endocrine-dependent diseases in later life [110,111,112]. Historically, most DOHaD-related research has been conducted in the prenatal period yet other critical windows include lactation, in which the child depends exclusively on one feeding mode, and adolescence. Exposure to environmental and dietary contaminants during any, some or all programming windows are likely to affect adult health status and increase risk of disease.

Human milk has the function to provide all dietary macro- and micronutrients and hundreds of non-nutritive bioactive molecules necessary for the infant’s survival, well-being and growth [4,5,6] Hence, it can be a vehicle for water- and fat-soluble contaminants at the same time. Despite contaminants being possibly present in human milk, the benefits of breastfeeding outweigh the potential health risk: Thus, mothers are generally encouraged to breastfeed for at least six months up to two years (Section 7) [1,2,3,113,114,115].

### 5.2. Mechanisms by Which Environmental and Dietary Contaminants Are Entering Milk

Most knowledge on how environmental and dietary contaminants gets into human milk is coming from pharmaceutical drugs, a specific subset of xenobiotics as reviewed in Ito and Lee [116]. Many environmental and dietary contaminants are transferred into human milk depending on their physico-chemical proprieties and their metabolic and excretion pathways. An overview of the water-and fat-soluble dietary and environmental contaminants found in human milk is provided in Table 4. Lipophilic small chemicals up to 500 Dalton with high plasma concentrations, poor protein-bonds or high pKa are overall those expected to be found predominantly in the human milk fat fraction [117,118,119].

However, these deposits are not eternal but depend on the integrity of the WAT. When lipolysis in adipose tissue is activated for example in periods of starvation, not only lipids but also lipophilic contaminants are mobilized and released from WAT. For some contaminants it has been shown that for every kilogram of weight loss their blood concentrations increase by 2–4% [142]. Together with the released lipids, contaminants are redistributed throughout the body mainly via very low density lipoproteins (VLDL). In women, the same effect occurs when the high energy demand late in pregnancy and during lactation requires mobilization of fat from adipocytes. Potentially, this leads to the release of large amounts of lipophilic contaminants from WAT that are co-transported with lipids to the mammary glands where they ultimately end up in milk. This mechanism has been elegantly measured in a rat model showing that the ^14^C-labelled model substrate 2,4,5,2′,4′,5′-hexachlorobiphenyl is released from fat stores late in pregnancy, transferred to VLDL and finally accumulates in the mammary glands [143]. This implies that even when nutrition during lactation is completely free of contaminants, the mother’s adipose tissue could still be an unavoidable source for them.

Besides WAT, bone tissues can also contribute to the rapid mobilization of contaminants to human milk. Toxic trace elements such as arsenic, cadmium, chromium, mercury and lead are efficiently absorbed by plants from the soil [144], accumulate in the mother’s skeleton and are released during the lactational period as it was demonstrated for lead [144,145]. Adequate calcium intake during pregnancy and lactation reduces this bone mobilization and with it the release of lead during lactation [146]. Overall and independently of the mechanism of transfer contaminants or their biologically active metabolites have the potential to reach concentrations that can be higher in human milk than those retained in the mother’s body [147].

We described in Section 3 how milk proteins and MFG are degraded during their way through the stomach and intestine. Although no human data are available, it can be expected that hydrophobic milk contaminants are associated with these vesicles or proteins. Consequently, they will be released during the solubilization process of the fat micelles and taken up by the infant in parallel with the other hydrophobic compounds.

### 5.3. Human Health Concerns of Environmental and Dietary Contaminants from Human Milk

A large body of evidence generated in experimental animal models [148] confirmed that critical programming windows sensitive to the exposure to environmental and dietary contaminants (DOHaD concept, Section 5.1) overlap with critical windows of exposure to EDs. Hence, environmental and dietary contaminants can interfere either with endogenous hormones or epigenetic regulators (e.g., histone deacetylases, DNA methyltransferases) during those critical growth and developmental phases affecting the pathophysiological status of infants with short- and long-term, possibly life-long [148] or even transgenerational, consequences [149]. For breastfeeding infants, human milk is at the same time an important source of nutrients, immuno- and bioactive molecules and a source of the infant’s exposure to environmental and dietary contaminants. These can be either fat- (e.g., the persistent organic pollutants, POPs, Section 5.4) or water-soluble (e.g., mycotoxins (Section 5.9) and toxic trace elements (Section 5.10 and highlighted in Table 4). Environmental and dietary contaminants are potentially responsible for food allergies and intolerances, antibiotic resistance, hormonal disturbances and poisoning [150]. Noticeably, some human milk contaminants behave unexpectedly such as the perfluorooctanoid acid (PFOA), a water-soluble chemical belonging to a fat-soluble class of POPs, the per- and poly-fluoroalkyl substances (PFASs, Section 5.7).

Short- and long-term consequences of the exposure to environmental and dietary contaminants that will be considered in the following sections include the interaction with bioactive molecules naturally occurring in human milk to whom antioxidant capacity has been attributed. Indeed, among the common targets of human milk bioactive molecules and xenobiotics, the “master regulator of antioxidant responses” Nrf2 (first mentioned in Section 4.1) plays a major role [78]. Environmental and dietary contaminants such as dioxin-like chemicals [151] (Section 5.4), mycotoxins [152,153,154] (Section 5.9) and arsenic [155] (Section 5.10) all affect Nrf2 expression and function. Their negative role could be blunted by the presence of antioxidant molecules in human milk (Section 4). Nrf2 activity is modulated directly or indirectly via endocrine-responsive or epigenetically-regulated proteins such as aryl hydrocarbon receptor (AhR), the so-called “dioxin receptor”, or Kelch-like ECH-associated protein (Keap) 1, respectively (Section 5.4). Furthermore, the potential interference of environmental and dietary contaminants with the endocrine system and with epigenetic regulators are taken into account in Section 5.4, Section 5.5, Section 5.6, Section 5.7, Section 5.8, Section 5.9 and Section 5.10.

### 5.4. Fat-Soluble Contaminants: Persistent Organic Pollutants

Persistent organic pollutants (PCBs) and dioxin-like chemicals but also organochlorine pesticides (e.g., DDT) are among the fat-soluble persistent organic pollutants (POPs) mostly studied in human milk [130,156,157,158]. Indeed, evidence of their presence and accumulation in human milk is consolidated and led to national and worldwide biomonitoring programs [159].

First concern about the risk human milk contaminated by dioxin-like chemicals came from the observation that the hypothalamic-pituitary-thyroid regulatory system was affected in newborns altering thyroid hormone levels [160]. Toxicological effects of these chemicals appear to be mediated by nuclear receptors such as the thyroid hormone receptors and AhR at low levels of exposure. Amongst others, they affect neuronal, immunological, metabolic, endocrine and reproductive tissues.

Importantly, several studies on the role of PCBs and dioxin-like chemicals on human health and on pesticides and plasticizers (Section 5.8) show that prenatal exposure to chemicals acting as antiandrogens were linked to male infertility and associated diseases [161,162]. This resulted in the conceptualization of the Testicular Dysgenesis Syndrome (TDS). TDS includes reproductive disorders of the newborn (the congenital birth defects cryptorchidism and hypospadias) and young adult males (low sperm counts and testicular germ cell cancer) and it is characterized by low levels of testosterone in the fetal testis [163]. In animal experimental models, it was interesting to note that similar TDS adverse effects can be also observed because of maternal exposure to EDs in the lactation period [163,164].

Exposure to POPs such as PCBs has been related to the development of inflammatory diseases such as atherosclerosis: PCB- and lipid-mediated induction of inflammatory genes and endothelial cell dysfunction occur as a consequence of increased cellular oxidative stress and an imbalance of the antioxidant status [165]. Interestingly, it has been proposed that α-tocopherol, flavonoids and ligands of the anti-atherogenic PPARs can protect against PCB-mediated endothelial cell damage [166].

Dioxin-like chemicals and other POPs affect Nrf2 expression and function via AhR, a xenobiotic chemical sensor that plays a role in multiple organs/tissues [167,168,169]. AhR is bound and activated by several xenobiotics and plant-derived bioactive compounds leading by complex signaling to high ROS production [151,167,168]. The activation by xenobiotics of such a nuclear receptor known to be involved in several inflammatory-based pathologies can be directly or indirectly attenuated by different plant-derived bioactive compounds acting as tissue-dependent AhR agonist or antagonists [168]. The indirect role of plant-derived bioactive compounds in preventing AhR-dependent ROS production is frequently mediated by Nrf2 activation and signaling via a well-established cross-talk with the AhR signaling [151,168,169].

Mechanistic in vitro studies have shown that xenobiotic-mediated activation of AhR in the mammary gland could be potentially harmful by two different mechanisms acting either directly or indirectly: in isolated mammary epithelial cells, dioxin-activated AhR impaired cell differentiation and, hence, possibly lactogenesis [170]. Indirectly, dioxin-activated AhR increased the gene and protein expression of the ATP-binding cassette (ABC) transporter ABCG2 in primary bovine mammary epithelial cells in a time- and dose-dependent manner which could result in an increased release of different xenobiotics into bovine milk [171].

### 5.5. Plant Protection Products

Plant protection products (PPPs) are a huge family of different classes of chemicals acting as biocontrol agents. Depending on their application they are known by the specific names of biocides, pesticides or herbicides and so on [172]. A large body of evidence associated adverse human health effects of several PPPs to endocrine-related diseases and the classification of each PPP as ED is taking a lot of efforts at the international level [173,174,175]. Most of the investigated hormone-related pathways are those mediated by the interaction of these xenobiotics with nuclear receptors controlling estrogen, androgen and thyroid signaling pathways—although many other mechanisms could be involved [176]. PPPs, in their role as anti-androgens, induce TDS as explained in Section 5.4, whereas others such as vinclozolin act both as an ED and via epigenetic modifications [177] (Section 5.1).

Similarly, to other POPs, the increased health risks to infants exposed to organochlorine pesticides arises from the incompletely formed detoxification mechanisms in infants that are potentially leading to an increased risk of developmental defects. Some endocrine disrupting organochlorine pesticides act as neurotoxins due to their ability to block the activity of inhibitory neurotransmitters, accumulate in the mammary gland and transfer to human milk during lactation [173,178]. A recent assessment of infant safety from 2020 associated human milk exposure to organochlorine pesticides and led to the conclusion that human milk reaching a concentration up to 6.81 ng/g lipids in the first month of lactation is still safe for infant health [178].

### 5.6. Polycyclic Aromatic Hydrocarbons

Polycyclic aromatic hydrocarbons (PAHs) such as benzo(a)pyrene (BaP) are mainly formed from the incomplete combustion of organic substances in coals, fossil fuels, oils and cigarettes [179]. PAH levels in human milk seem to be associated with the air pollution level in the maternal place of residence where they are present as air pollutants [57]. PAHs have been associated with cancer, immunological-, cardiovascular- and respiratory-linked pathologies but also to fetal growth impairment. Furthermore, and similar to dioxin-like chemicals (Section 5.4), BaP is impinging on the AhR and Nrf2 signaling pathways in vitro [151,167] and also on Nrf2 itself. As recently reviewed in 2020, mechanisms of BaP-induced tumorigenesis appeared to be mediated by epigenetic regulations at the level of miRNA expression [180]. The latter being proposed by the same authors as a common theme for several xenobiotic-induced cancers.

Although not completely established [57], breastfeeding has protective effects against outcomes of indoor and outdoor air pollution exposure (i.e., either to PAHs and/or particulate matters), immunological-, cardiovascular- and respiratory-linked pathologies, and decreases under-five years mortality in both developing and developed countries that are overall contributing to the benefits of breastfeeding (Section 7.1). Such breastfeeding protective effect has been related to the immunomodulatory, anti-inflammatory, antioxidant and neuroprotective properties of human milk components such as those listed in Table 2 (Section 4).

### 5.7. Per- and Polyfluoroalkyl Substances

Per- and polyfluoroalkyl substances (PFASs) are POPs ubiquitous in the environment particularly in drinking water. These are xenobiotics of emerging concerns; they have caught research and public health attention only in recent years [181]. The most known and studied PFAS is perfluorooctanoid acid (PFOA), a chemical accumulating more easily in blood serum than in fat tissues. PFOA has been recently shown to be present in several biological matrices including human milk [181,182]. Both breast- and formula-fed infants are a sensitive subpopulation for PFOAs’ developmental interference. Their exposure is higher per body weight than in adults and PFOAs enter their food chain through formula prepared with contaminated drinking water or via human milk [181].

Early life exposure to PFASs in human milk has been correlated in 2020 to different negative outcomes in infants, although further studies are necessary to confirm the first and so far weak evidence from very few studies [183]. A European-based meta-analysis including more than 4800 mother–child dyads suggested an increased prevalence of attention-deficit/hyperactivity disorder (ADHD) linked to PFAS exposure in girls, in children from nulliparous women and in children from low-educated mothers [183]. A small Chinese cohort of 174 mother-infant pairs correlated PFAS exposure via human milk and negative outcomes in postnatal growth, particularly infants’ length and weight gain rates [127].

### 5.8. Plasticizers

Plasticizers are plastic additives or constituents of plastics. Among them, phthalates and bisphenols are well known due to their application as softener in polyvinyl chloride (PVC)-based and polycarbonate plastics and epoxy resins, respectively. Phthalates are diesters of phthalic acid (1,2-benzenedicarboxylic acid) and are synthetic organic chemicals used not only as plasticizers but also in industries as solvents, personal care products and medical devices, in particular blood bags and tubing for oxygenation or enteral nutrition. Indeed, the chemical mostly used in medical devices, di(2-ethylhexyl) phthalate (DEHP), ensures not only flexibility but also durability. It is added up to 50% (by weight) in some devices such as blood bags [93,184]. Bisphenols are diphenols largely used in food contact materials: They are used either as a building block in polycarbonate plastics or as a precursor of epoxy resins in the internal layer of metallic containers such as cans [185]. The most known representative of this class is bisphenol A [BPA, 4,4′-(propane-2,2-diyl) diphenol)], one of the highest-volume chemicals produced worldwide.

Both phthalates and bisphenols are known or suspected EDs acting as antiandrogens or estrogen-like chemicals, respectively. Some of them are among the few substances of very high concerns officially recognized by the European Chemicals Agency (ECHA) as endocrine-disrupting chemicals [186]. Chemicals in both classes are also indicated as obesogenic EDs or obesogens since their effects are recognized to contribute to obesity and other metabolic disturbances [185,187,188,189,190,191]. Noticeably, in human milk, an average BPA concentration of 0.61 ng/mL has been reported, whereas phthalates are detected as metabolites in this matrix only [120].

Phthalates and bisphenols are known to act via multiple nuclear receptors such as thyroid and sex steroid hormone receptors, in particular thyroid receptors (TRs), the androgen receptor AR, estrogen receptors ERs and the so-called ER-related (ERRs) receptors but also PPARs and others. Moreover, it has been shown in experimental models that (i) vitamin D bound to its receptor VDR is able to prevent immunological adverse effects of BPA exposure when maternally supplemented [191] and (ii) that α-tocopherol prevents adverse reproductive outcomes in a PPAR-dependent manner when supplemented in the perinatal period [192]. Far to be demonstrated in humans, the last two studies contribute to shed light on the molecular mechanisms linking low vitamin (antioxidant) levels or even deficiencies on endocrine-regulated pathways when an unwanted exposure to environmental and dietary contaminants occurs in the perinatal and lactational periods.

To add further complexity in such multiple interactions, it was recently shown in 2020 that perinatal exposure of rats to BPA affected endocrine-related outcome (i.e., anogenital distance, testis weight, serum testosterone concentrations), expression of antioxidant-associated enzymes (i.e., SOD, GST and GSHPx concentrations) and epigenetic marks, such as the hypermethylation of the ERα at the same time [193]. A direct BPA mechanistic role in regulating the antioxidant-associated enzymes has been so far suggested by the discovery that Nrf2-mediated signaling is activated in vitro during the first steps of placentation [194,195], whereas prenatal and lactation BPA exposure in vivo has been shown to be linked to hepatic steatosis in mouse offspring [195]. Interestingly, in the latter case, Nrf2 activation has been likely associated to a novel function independent from its known antioxidant role as a direct regulator of enzymes and transcription factors inducing de novo lipogenesis. Overall, a paucity of data regarding the exposure to phthalates and bisphenols during lactation exists. To the authors’ knowledge only one study investigated this topic in rodents [195] and further investigation is needed.

### 5.9. Water-Soluble Contaminants: Mycotoxins

Mycotoxins are relevant xenobiotics since consumption of mycotoxin-contaminated ingredients are increasing worldwide because of climate change and leading to their broader presence in animal foodstuffs such as meat, eggs and animal [196] and human milk [197]. In adults, concerns about mycotoxin ingestion is mainly due to their carcinogenic, mutagenic and genotoxic effects as well as on the endocrine disrupting effects on reproductive tissues. In infants, the main concern relies on a more general aspect: following consumption, mycotoxins are absorbed by the upper part of the intestine [198,199]. Hence, the intestinal epithelial barrier represents the first defensive barrier towards mycotoxins. Although not yet demonstrated in humans, experimental models revealed a role of mycotoxins in alterations of several intestinal functions such as digestion, absorption, permeability, defense and, although questionable, on gut microbiota composition as well. A direct effect on decreasing secretion of molecules such as mucins and activation of cytokine production has also been indicated [196,200,201]. Overall, these events are directly involved in the intestinal physical, chemical, immunological, microbial integrity and general homeostasis of the barrier (reviewed in [196]). Despite this, Tesfamariam and colleagues aimed to highlight the association between dietary mycotoxins exposure and growth and morbidity of children aged five years or younger. Their systematic review from 2020 did not rule out a possible association because of the low overall quality of the published studies [202].

### 5.10. Water-Soluble Contaminants: Toxic Trace Elements

Among water-soluble contaminants in human milk trace elements classified as toxic (Section 4.3) such as lead, mercury, cadmium and arsenic are among the most relevant since they are being detected worldwide and rising [138]. Exposure to these toxic trace elements in general from air, drinking water and diet [138] during critical programing windows (Section 5.1) is feared because of susceptibility especially of the central nervous system [203]. However, it is difficult to distinguish later recognized adverse outcomes from prenatal versus postnatal exposure, and separate impact through pregnancy to lactation or the period of rapid infant growth during the first year of life. Concerns are strengthened because intake of toxic trace elements may be proportionally higher per body weight compared to adults, due to increased gastrointestinal absorption coupled with a lower renal excretion capacity and body clearance [204]. It is important to state in this context that the potential risks to infants exposed to toxic trace elements are outweighed by the benefits of human milk consumption (Section 7.1) [138].

Lead can enter the body storage pool and accumulate over time. During lactation, maternal bone minerals are released and enrich milk particularly with calcium (Section 5.2) but also with lead [146,205]. Especially in utero, the central nervous system is a critical target for lead exposure: adverse effects include intellectual and behavioral deficits, hyperactivity, fine motor function deficits, decreased intelligence quotient, alteration of hand-eye coordination and problems in reaction time [205]. Human milk concentrations between 2.0–5.0 μg/L were suggested as acceptable reference values [205].

Another trace element with prenatal impact on neurocognitive function is mercury. Mercury does not accumulate in human milk per se and its concentrations are generally lower than those of lead and about three times lower than in maternal plasma [146]. Highest levels for mercury are associated in adults with high fish consumption [138] However, in the form of methylmercury it is easily absorbed in the gastrointestinal tract and passes the blood-brain barrier [206]. After the accident in Minamata bay in Japan in 1956 after which mothers consumed methylmercury contaminated foods with higher concentration than those reported today breastfed infants were negatively affected [146,207].

Cadmium concentrations in human milk are generally linked to maternal smoking before and during pregnancy, secondhand exposure and maternal postnatal fish consumption [146,208]; concentrations are about 35% of those in maternal blood [208]. Transition of cadmium into human milk may be facilitated by some fatty acids (specifically, oleic, elaidic and *cis*-vaccenic acids) [208].

Chronic exposure to arsenic has been linked to increased risk for numerous cancer types, skin lesions, reduced fertility and altered childhood cognitive function [209]. However, direct exposure via breastfeeding is still seen as negligible [210,211]: Even though maternal exposure through food or drinking water may be high, little of this translates into human milk or transfers to the infant [137]. Daily intake from exclusive breastfeeding corresponds to 0.01–0.17 μg/kg body weight [212] and human milk concentrations in the range of 1–25 μg/L are considered safe [39]. Arsenic is expelled in urine in its methylation form(s) and this mechanism is functional in infancy [137]. Another detoxification mechanism is the conjugation of arsenic and mercury through GSH through action of GST, emphasizing the importance of these enzymatic systems [39].

## 6. Food Safety and Monitoring Aspects

Most of the environmental and dietary contaminants listed in Table 4 have been investigated as potential or known EDs. Most of the evidence for adversities of exposure to many EDs are coming from different in vitro and in vivo experimental models. In humans, proving adverse effects of these contaminants is more difficult unless critical events happen such as the mass-release of methylmercury that occurred in Minamata bay, Japan, in 1956 [207]. Hence, human health risk is generally determined by evidence coming from experimental animal models. Under the European REACH regulation [213] only a few contaminants, namely five phthalates and BPA, are officially listed as substances of very high concern by ECHA because of their endocrine-disrupting properties [186].

The exposure risk to contaminants has been approached by total diet studies specifically to evaluate dietary exposure to chemical substances assessing the efficacy of risk management strategies in the prevention of public health issues [214,215]. Estimating chemicals’ intake is commonly approached by merging data of contaminant concentrations and food intake [216]. However, for total diet studies the estimation of chemical intake is difficult because of food variation and differences in dietary preferences. The first six months simplifies biomonitoring because exclusive breastfeeding is the only food source [159,217]. Maintaining and even broadening human biomonitoring in milk should be performed to keep milk and breastfed infants as safe as possible, also considering that substituting formulas encounters similar troubles, in particular when powders are reconstituted in the household with possibly contaminated tap water in which organic chemicals are eventually not even monitored.

In Europe, risk assessment on chemicals that can be present in food and feed is performed by the European Food Safety Authority (EFSA)’s “Panel on Contaminants in the Food Chain” [218]. In the past, the panel’s activities covered mostly organic xenobiotics (e.g., POPs such as dioxin-like chemicals and brominated flame retardants) or trace elements (e.g., nickel, arsenic and chromium among the most recently evaluated). EFSA considers exposure contribution by human milk in their scientific advice. To apply this approach to human milk, the completion of human biomonitoring programs prioritizing human milk as food matrix should be available as in progress, for instance, at the European level by a human biomonitoring project involving 30 countries: the European Environment Agency and the European Commission [219]. In parallel to monitoring, WHO and medical associations in Europe and North America recommend breastfeeding as discussed in the next chapter.

## 7. What Is Known of Benefits vs. Risk of Breastfeeding under Consideration of Environmental and Dietary Contaminants

The Centers for Disease Control and Prevention (CDC) as part of the United States Department of Health & Human Services states that although human milk contamination is a known issue, breastfeeding is recommended and fully endorsed. Cut-off levels for environmental and dietary contaminants that would help clinical interpretation are largely missing and human milk composition is not routinely monitored (yet). The CDC explains that adverse effects were only observed in breastfeeding infants when the mother’s health was severely compromised through toxic exposure [113]. Generally, negative effects are associated with acute poisonings or extreme situation [115], which then explains that medical associations such as the AAP and ESPGHAN also endorse breastfeeding because the associated benefits outweigh the risks [2,3].

The WHO and United Nations Environment Programme run several programs aiming to improve mother and child health through environmental safety and address, besides other factors, actions to reduce air pollution especially from indoor cooking, drinking water contamination and other environmental hazards [220]. They recognize the risk of human milk contamination for the child. However, human milk contamination presents a minor part in relation to direct exposure. They conclude from their “human milk contamination surveys” of 2017 that “the risk-benefit debate of breastfeeding may as well be redundant” because infants experienced only subtle—often transitory—adverse effects from breastfeeding [159]. Discontinuation of breastfeeding under these circumstances would reduce the contamination burden insignificantly and deny the child the experience of the many benefits associated with human milk consumption (see below) [114]. In contrast to EFSA’s human milk monitoring endeavors described in chapter 5, their recommendation for future risk-benefit studies is to focus on the critical prenatal window, instead [159].

### 7.1. Breastfeeding Benefits

Breastfeeding is sustainable, immediate and cheap and its preparation does not require specific tools made from plastic or an external water source [114,221]. Breastfeeding benefits go beyond providing nutrients and beyond benefits for the child alone: Economic advantages have been calculated in the past to equal more than 300 billion U.S. dollars and, more recently, up to 341 billion U.S. dollars [222,223]. Improvement to societies are associated with higher intelligence scores from breastfeeding, subsequent higher household incomes and generally saving more than eight hundred thousand lives annually [48]. More directly and shortly after birth, the mother-infant dyad benefits from bonding induced by oxytocin-release from close proximity and skin-to-skin contact [224]. Oxytocin also contracts the uterus after birth and reduces maternal blood loss associated with birthing [1]. Long-term, mothers that breastfed are less likely to develop ovarian, epithelial ovarian, endometrial and breast cancers [225,226] and are less at risk for metabolic syndrome, cardiovascular disease, hypertension and diabetes mellitus type 2 [225,227,228]. These benefits are correlated positively to breastfeeding duration [48,225]

Research on long-term breastfeeding benefits for the child are riddled with confounders. It is impossible to conduct a randomized clinical study because a mother knows whether she is breastfeeding or not. In addition, there are many lifestyle factors that cannot allow control such as educational status or intelligence scores, socioeconomic standing, exposure to environmental or dietary contaminants, the degree of health of maternal diet, smoking and secondary smoke exposure or the general better quality of life that is observed in women who decide to breastfeed [229]. Despite these limitations, it is well established that breastfed children end up with higher intelligence scores [48] from which societies can prosper [223]. Many short-term benefits are linked to the many bioactive compounds in human milk and especially in colostrum (Box A1, Table 1). They have trophic, antioxidant (Section 4, Table 2), anti-inflammatory and probiotic properties, and contribute to human milks’ immuno-protective effects and a significant overall reduction of mortality in the first five years of live [48]. This “survival through breastfeeding” is the result of reduced risks for (infectious) diarrhea, gastroenteritis, respiratory and acute ear infections, the need and lengthy duration of hospitalization and the sudden infant death syndrome [2,48].

### 7.2. Breastfeeding Discontinuation

The AAP describes some situations (e.g., compromised maternal health with radiation therapy or (chemo)therapeutic agents) in which it could be considered to discontinue breastfeeding or where expressed milk seems to be the preferred (maybe temporary) way forward [2]. With regard to environmental and dietary contaminants in human milk (Section 5) and the consideration for a decision of breastfeeding discontinuation, individual assessment may be necessary for those instances that human milk contamination has reached critical levels and presents in clinical symptoms of mother or the child. In these extreme and rare cases, also the WHO suggests considering reducing breastfeeding duration to less than the ideal six months of exclusive breastfeeding and fast interventions towards elimination or reduction of the (environmental) risk [114].

As described in Section 6, the general issue remains that unified actions and monitoring need to take place because human milk contamination cannot be addressed on an individual basis [230]. Monitoring programs for human milk have been suggested or implemented as well as calls to action to governments, societies and health authorities securing drinking water quality and regulating the other pathways that environmental and dietary contaminants affect human health globally [111,114,138,159,219,220,230,231].

### 7.3. How to Reduce Individual Exposure

Despite the predominant factors that affect human milk contamination being out of the individual’s control, mothers and families can take some steps to reduce their child’s exposure. Exclusive breastfeeding for at least six months and an extended breastfeeding duration are two of these steps: Since some of the toxic trace elements (Section 5.10) do not seem to translate strongly into human milk, exclusive breastfeeding reduces exposure through other channels [115,137]. With mixed or formula-feeding drinking water quality and also that of feeding tools made from plastic or rubber used for the product preparation can potentially play roles to increase exposure and should be avoided if these are used and it is possible to remove [138,211].

Neurotoxicological effects can also be countered through breastfeeding because human milk contains many factors that support neurocognitive development. These are held responsible as to the reason that breastfed infants have higher intelligence scores [48,115]. In addition, mothers can further strengthen this effect through fish intake and docosahexaenoic acid (DHA) consumption or supplementation. EFSA performed a benefit-to risk assessment about fish and seafood consumption in the presence of methylmercury [99]. They concluded that one to four servings of fish and seafood per week are needed to reach the dietary reference value for omega-3 LCPUFA. These fatty acids translate into milk and are associated with neurobehavioral benefits in children. In case of high seafood contamination, the number of fish and seafood servings should be reduced to one or two per week to stay below the tolerable weekly intake of methylmercury. This assessment is in line with the earlier recommendation of the AAP, which suggests that well-nourished breastfeeding mothers who want to increase their intake of omega-3 long-chain polyunsaturated fatty acids eat one to two portions of fish per week to reduce mercury and cadmium intake. However, predatory fish such as pike, marlin, mackerel, tile fish and swordfish should be avoided to reduce excessive mercury intake or that of other dietary contaminants from these food sources [2].

Fast changes of body weight, i.e., adipose tissue activation should be carefully monitored if not avoided: the environmental and dietary contaminants stored in WAT will be mobilized and secreted with human milk (Section 5.2). The mothers’ age is here a factor because an older woman with high body mass index is more likely to have stored higher contaminant levels in her WAT. Maintaining weight before and during pregnancy and during lactation has been advised. Reducing intake of fatty foods from animal origin and avoiding disproportional weight gain during pregnancy has also been suggested as countermeasure [115].

Similarly, bone minerals are activated during lactation (Section 5.2), which can cause release of lead stored in this tissue. Intake of sufficient calcium concentrations or supplementation can be considered as countermeasure especially if mothers live in areas where environmental lead levels are high [146].

Use of recreational drugs and alcohol are not seen as contraindications to breastfeed nor is nicotine and secondary smoke exposure although it is known that cadmium concentrations in human milk increase through smoking and that alcohol reduces milk flow [2,146]. Mothers and their families that are concerned about substances with poorly investigated effects transferring through milk into the child should consider changing her and her family’s lifestyle and receive full support to implement these changes [115].

## 8. Perspectives

### 8.1. A Role for Epigenetics

In our review we have so far focused mainly on human milk composition, contaminants in human milk, their interaction with antioxidants and the acute consequences for the infant. This neglects our insights that the early environment also induces long-term and sometimes even permanent alterations in the organism. This concept is now generally accepted as the DOHaD paradigm (Section 5.1) [110]. Mechanistically, it includes gross changes in organ structure and morphology but also molecular changes, especially epigenetic modifications [110].

Best studied in this respect are long-lasting epigenetic marks encoded as changes in DNA methylation. The general idea here is that early environmental factors can act on the DNA methylation machinery, which leads to changes in methylation at CpG dinucleotides. These can than either influence gene expression permanently or be present as methylation marks on the DNA without further known function [232]. Historically, the first studies on epigenetic changes induced by environmental factors are related to maternal nutrition, e.g., undernutrition as shown in the Barker studies or the Dutch hunger winter cohort [166]. Since then the field has broadened to include maternal stress, overnutrition, pregnancy disorders and—relevant for this review—toxic compounds in the diet or (secondary) cigarette smoke exposure. Moreover, also the duration of exposure has been extended: current information on the lactation period as a critical window has been summarized by Pico and colleagues in 2020 [112].

Amongst the toxic compounds, the best studied candidate is BPA, which has been shown to stimulate the development of metabolic dysfunction in animal models by epigenetic mechanisms (see [233,234] for review). Several of the factors identified in animal studies have subsequently been also found in human samples. Key examples are insulin-like growth factor 2 (IGF2) and PPARα [235]. Another interesting factor is Nrf2, which was introduced before as one of the major players of antioxidant response (e.g., Section 4.3, Section 4.4 and Section 5.3). The gene encoding Nrf2, *NFE2L2*, is both directly and indirectly under epigenetic control. Direct epigenetic regulation occurs by methylation of the *NFE2L2* gene promoter. Indirect regulation occurs via epigenetic modulation of upstream factors, e.g., Keap-1, which will subsequently change Nrf2 levels in the cell [78]. Moreover, also miRNAs are known to regulate the transcripts of these upstream factors. These few examples of epigenetic modification are just the tip of the iceberg of the many changes that are potentially induced by contaminant exposure early in life.

### 8.2. A Role for Microvesicles and miRNAs

Since the description of functional transfer of RNA—especially miRNAs—as a means of communication between cells by Valadi and colleagues [236], this exosome- or vesicle-mediated miRNA transfer has been implicated in many processes in the body. Currently, extracellular vesicle-bound miRNAs are considered to be more important for information transfer than free RNA present in many body fluids [237].

Not surprisingly, human milk also contains specific and characteristic microvesicles that are loaded with miRNAs mainly derived from the mammary gland [238]. Based on the assumption that in milk these vesicle serve as messengers, Melnik and Schmitz proposed that the breastfeeding mother has a means to influence gene expression in her child’s intestinal cells and beyond through milk miRNA [239]. This could let the mother actively manipulate absorptive processes in the child, possibly and specifically adapted to her milk’s composition and her child’s need. Recently, Carrillo-Lozano and colleagues summarized in 2020 current knowledge on this hypothesis but concluded that the evidence is not yet completely convincing [240]. Gaining a better understanding of these processes should be the target of future investigations.

### 8.3. A Role for the Microbiota

Hydrophobic contaminants are released during digestion (Section 3) from MFG and proteins in the stomach and early in the intestine. If not immediately absorbed, they will then be potential substrates for the intestinal microbiota. The evaluation of the effects of contaminants is complicated by the neonatal microbiota, which is different from that in adults. Neonatal bile acids can be used as example for the development of complex molecule metabolism: the neonatal bile acid pool is rather small and consists predominantly of primary bile acids because the bile acid metabolizing bacteria are either not yet present in large populations or are not yet active [241]. Similarly, it can be speculated that bacteria acting on organic contaminants of the diet are not yet active in neonates. This could imply that for some compounds, bacterial degradation and therefore detoxification is less prominent than in adults. On the other hand, it could also mean that activation of some other compounds by bacteria is absent. Together, there is currently not enough data available to decide whether the early microbiota leads to advantages or disadvantages with respect to toxicity of contaminants and their metabolites.

### 8.4. A Role for Human Milk Research

Human milk composition is highly variable. For a better understanding of its composition relevant also for the ongoing food safety and monitoring programs described in Section 6, comparability of outcomes is required. This affords development and compliance to international standardized protocols for milk collection, storage, methodology and analysis. The entirety of antioxidative bioactive molecules and that of many contaminants in milk remains unknown and therefore the impact of these molecules and/or their bioactive metabolites on health remains largely unexplored. Interactions of milk bioactive molecules with signaling pathways and transcription factors or environmental and dietary contaminants within the complex matrix that is milk should be further explored because a deeper understanding of these interactions will support the application of the DOHaD concept (Section 5.1) and subsequent prevention of immediate, later and lasting disease.

## 9. Conclusions

Breastfeeding is the ideal nutritional choice for any infant [1,242]. This recommendation is not compromised when considering potential milk contamination as the benefits of breastfeeding outweigh the risks [113,159]. We here presented a compilation of human milk composition considering the different lactation stages. Milk fractions, formation of the milk fat architecture and the distribution of some antioxidative molecules are explained. We also give an overview about typical environmental and dietary contaminants, which often can be found in milk samples and some of their properties.

There are many publications characterizing the toxic effects of these contaminants for the human body although their role upon early exposure (i.e., during lactation) is limited. Here we add a short description of their role as endocrine disruptors and their potential long-term epigenetic effects. Monitoring efforts and recommendations for breastfeeding mothers are included. Finally, we speculate that the still developing microbiota of the infant might undergo poorly defined interactions with milk contaminants, leaving the necessity to perform more studies in future.

## Figures and Tables

**Table 1:** Selected bioactive molecules and minerals in human milk. Various sources and lactation stages describe the high variation in human milk. “Milk” stages are explained in Appendix A, Box A1. The general term “milk” refers to non-specified lactation stages in the source.

Molecule and Lactation Stage		Unit and Value	Source
**Vitamin A**		μg retinol ester/L	
Milk	range	450–600	[7]
Colostrum	range	203–2000	[8]
Transitional milk	range	132–2050	[8]
Mature milk	range	137–1080	[8]
Mature milk	range	300–600	[9]
**Carotenes and carotenoids**		μg/L	
Colostrum	mean ± SD	2000 ± 120	[8]
Colostrum	mean	2180	[9]
Transitional milk	mean ± SD	1000 ± 40	[8]
Mature milk	mean ± SD	230 ± 50	[8]
*α-carotene*		nmol/L	
Colostrum	mean ± SEM	59.0 ± 13.5	[10]
Mature milk	range	10–14	[11]
Mature milk	mean ± SEM	19.2 ± 3.0	[10]
*β-carotene*		nmol/L	
Milk	median	49.4	[12]
Milk	mean	32.8	[13]
Colostrum	range	125–423	[12]
Colostrum	mean ± SEM	164.3 ± 25.2	[10]
Mature milk	range	18–78	[12]
Mature milk	range	36–50	[11]
Mature milk	mean ± SEM	104.4 ± 27.7	[10]
*Lutein*		nmol/L	
Milk	median	114.4	[12]
Colostrum	range	69–280	[12]
Colostrum	mean ± SEM	121.2 ± 20.9	[10]
Mature milk	range	27–88	[12]
Mature milk	mean ± SEM	61.9 ± 10.9	[10]
*Zeaxanthin*		nmol/L	
Colostrum	range	10–33	[12]
Colostrum	mean ± SEM	46.3 ± 5.4	[10]
Mature milk	range	6–20	[12]
Mature milk	mean ± SEM	22.8 ± 2.7	[10]
*β-cryptoxanthin*		nmol/L	
Milk	median	33.8	[12]
Colostrum	range	61–238	[12]
Colostrum	mean ± SEM	57.4 ± 10.7	[10]
Mature milk	range	17–61	[12]
Mature milk	range	17–25	[11]
Mature milk	mean ± SEM	27.5 ± 4.8	[10]
*Lycopene*		nmol/L	
Milk	mean	85.9	[13]
Milk	median	33.7	[12]
Colostrum	range	137–508	[12]
Colostrum	mean ± SEM	119.9 ± 18.9	
Mature milk	range	19–60	[12]
Mature milk	range	19–35	[11]
Mature milk	mean ± SEM	68.0 ± 16.3	[10]
**Vitamin B6** pyridoxal-5′-phosphate		μg/L	
Milk	mean	130	[7]
Milk	mean	150	[14]
Milk	range	81–414	[14]
**Vitamin C**		mg/L	
Milk	range	35–90	[7]
Colostrum	range	39–190	[8]
Transitional milk	range	42–180	[8]
Mature milk	mean ± SD	38 ± 35	[8]
**Vitamin E**		mg α-tocopherol equivalents/L	
Milk	mean	3.50	[7]
Colostrum	mean ± SD	10.13 ± 1.50	[15]
Transitional milk	mean ± SD	4.59 ± 0.93	[15]
Mature milk	mean ± SD	3.00 ± 0.85	[15]
*α-tocopherol*		mg/L	
Colostrum	mean ± SD	9.99 ± 1.51	[15]
Colostrum	range	8.86–24.55	[16]
Colostrum	mean ± SD	11.00 ± 2.50	[8]
Transitional milk	mean ± SD	4.45 ± 095	[15]
Transitional milk	range	2.60–16.36	[16]
Mature milk	mean ± SD	2.92 ± 0.84	[15]
Mature milk	range	2.70–9.84	[15]
Mature milk	range	1.00–9.84	[16]
*γ-tocopherol*		mg/L	
Colostrum	mean ± SD	0.57 ± 0.21	[15]
Transitional milk	mean ± SD	0.60 ± 0.21	[15]
Mature milk	mean ± SD	0.30 ± 0.14	[15]
Mature milk	range	0.25–1.17	[15]
**Choline** (water soluble)		μmol/L	
Mature milk	mean (95% CI)	1102 (1072, 1133)	[17]
		mg/L	
Colostrum	mean	70	[7]
Mature milk	mean	160	[7]
Mature milk	range	57–318	[7]
**Ubiquinone**		μmol/L	
Milk	mean ± SD	0.32 ± 0.21	[18]
Colostrum	mean ± SD	0.81 ± 0.06	[19]
Transitional milk	mean ± SD	0.75 ± 0.06	[19]
Mature milk	mean ± SD	0.54 ± 0.33	[19]
**Selenium**		μg/L	
Colostrum	range	14–83	[20]
Colostrum	mean	41.0	[20]
Transitional milk	median	19.8	[21]
Mature milk	range	3–283	[20]
Mature milk	range	10–30	[20]
Mature milk	range	3–84	[7]
Mature milk	mean ± SD	16.3 ± 4.7	[7]
**Zinc**		mg/L	
Colostrum	range	8–12	[20]
Transitional milk	mean	3.66	[22]
Mature milk	range	1–3	[20]
Mature milk (4 weeks)	mean ± SD	4.11 ± 1.50	[7]
Mature milk (1–2 month)	mean ± SD	1.91 ± 0.53	[7]
Mature milk 3–5 month	mean ± SD	0.98 ± 0.35	[7]
Mature milk (6–11 month)	mean ± SD	0.77 ± 0.22	[7]
Mature milk	median	0.625	[22]
**Copper**		μg/L	
Colostrum	range	500–800	[20]
Transitional milk	median	590	[22]
Mature milk	range	200–400	[20]
Mature milk	mean	329–390	[7]
Mature milk	median	368–400	[7]
**Manganese**		μg/L	
Colostrum	range	5–12	[20]
Mature milk	range	3–6	[20]
Mature milk	range	3–30	[7]
Mature milk	mean	4	[7]
**Iron**		mg/L	
Transitional milk	median	0.46	[22]
Mature milk	mean	0.88	[22]
Mature milk	range	0.20–0.80	[20]
Mature milk	range	0.20–0.40	[7]
Mature milk	range	0.20–0.50	[22]
**Magnesium**		mg/L	
Colostrum	range	2.29–4.41	[23]
Mature milk	range	15–64	[7]
Mature milk	median	31	[7]
Mature milk	range	2.4–3.58	[23]
**Thioredoxin**		μg/L	
Colostrum	mean ± SEM	268 ± 23	[24]
**α-lactalbumin**		g/L	
Colostrum	mean ± SD	4.56 ± 0.41	[25]
Transitional milk	mean ± SD	4.30 ± 1.19	[25]
Mature milk	mean ± SD	2.85 ± 0.24	[25]
**Ceruloplasmin**		mg/L	
Colostrum	mean ± SD	150 ± 30	[26]
Transitional milk	mean ± SD	40 ± 20	[26]
**Lactoferrin**		g/L	
Colostrum	range	6–8	[27]
Colostrum	range	5–6	[28]
Colostrum	mean ± SD	3.53 ± 0.54	[29]
Colostrum	mean ± SD	6.15 ± 0.89	[25]
Colostrum	mean	5	[30]
Transitional milk	mean ± SD	3.65 ± 1.19	[25]
Mature milk	range	1–2	[27,28]
Mature milk	mean	2	[30]
Mature milk	mean ± SD	1.76 ± 0.28	[25]
Mature milk (6–14 weeks)	mean ± SD	1.65 ± 0.29	[29]
Mature milk (14–26 weeks)	mean ± SD	1.39 ± 0.26	[29]
Milk	range	1.2–3.1	[30]
**Osteopontin a**		mg/L	
Milk	mean	130	[31]
Milk	range	60–220	[30]
Milk	mean ± SD	128 ± 79	[30]
Colostrum	mean ± SD	180 ± 100	[25]
Mature milk	mean ± SD	138 ± 90	[25]
**Erythropoietin**		units/L	
Milk	mean	0.8–4.1	[32]
Milk	median	4.1–4.7	[32]
Milk	mean ± SD	11.7 ± 0.75	[33]
Transitional milk	mean	9.4–12.0	[33]
Mature milk	mean ± SD	33.8 ± 6.14	[33]
**Melatonin**		pg/ml	
Daytime milk	IR	1.5 (1.0–2.1)	[34]
Nighttime milk	IR	7.3 (3.8–13.6)	[34]
Nighttime colostrum	mean	25.3–28.7	[35]
Nighttime transitional milk	mean	22.6–24.7	[35]
Nighttime mature milk	mean	20.1–22.4	[35]
**Glutathione peroxidase activity**		units/L	
Colostrum	mean ± SD	73 ± 21	[36]
Transitional milk	mean ± SD	75 ± 24	[36]
Mature milk	mean	68–80	[36]
Milk	mean ± SD	73 ± 21	[36]
Mature milk	mean	31–77	[37]
Mature milk	mean ± SD	51 ± 15	[23]
Mature milk	range	25–80	[20]
Mature milk	range	31–39	[38]
Mature milk	mean	90.8	[21]
		units/mg protein	
Colostrum	mean ± SD	0.08 ± 0.04	[36]
Transitional milk	median	0.04–0.08	[22]
Transitional milk	median	0.06	[21]
Transitional milk	mean ± SD	0.15 ± 0.05	[36]
Mature milk	mean	0.08–0.16	[36]
Milk	mean ± SD	0.10 ± 0.04	[36]
**Glutathione reductase activity**		units/mg protein	
Transitional milk	median	0.01–0.02	[22]
Transitional milk	median	0.02	[21]
**Glutathione S-transferase activity**		units/mg protein	
Transitional milk	median	0.001–0.003	[22]
Transitional milk	median	0.002	[39]
		nM/min/mg protein	
Colostrum	mean ± SD	34.03 ± 5.76	[40]
Mature milk	mean ± SD	56.13 ± 10.59	[40]
**Superoxide dismutase activity**		units/mg protein	
Colostrum	mean ± SD	4.0 ± 2.9	[36]
Transitional milk	mean ± SD	5.6 ± 2.8	[36]
Transitional milk	median	132.4–267.0	[22]
Transitional milk	median	198.2	[21]
Mature milk	mean	4.3–5.6	[36]
Milk	mean ± SD	4.8 ± 2.0	[36]
**Catalase activity**		units/mg protein	
Colostrum	mean ± SD	0.50 ± 0.08	[41]
Transitional milk	mean ± SD	0.72 ± 0.10	[41]
Transitional milk	median	0.23–0.27	[22]
Transitional milk	median	0.25	[21]
Mature milk	mean ± SD	0.97 ± 0.21	[41]
**Glutathione**		μmol/L	
Transitional milk	mean ± SD	252.5 ± 173.9	[42]
Mature milk	mean ± SD	164.9 ± 128.0	[42]

“Range” describes the range of mean values reported in the cited source from multiple studies. “Mean” describes several means from the same study.

**Table 2:** A subset of human milk components with reported direct or indirect (anti)oxidative capacities by acting as radical scavengers, modulate enzyme activity, enzyme expression or interact with transcription factors and/or signaling pathways. Only a few components in human milk that demonstrate antioxidant capacity have been identified so far [22,38,41,49,57,66,67,68,69,70,71].

Enzymatic Antioxidants	
	glutathione peroxidase (GSHPx, E.C.1.11.1.9) glutathione reductase (GR, E.C.1.6.4.2) glutathione S-transferase (GST, E.C.2.5.1.18) superoxide dismutase (SOD, E.C.1.15.1.1) catalase (CAT, E.C.1.11.1.6)
**Non-Enzymatic Antioxidants**	
Enzymatic cofactors	
*Quinone*	ubiquinone (coenzyme Q10)
*Minerals*	selenium zinc copper manganese iron
Bioactive molecules	
*Vitamins*	α-tocopherol (vitamin E) ascorbic acid/dehydroascorbic acid (vitamin C) pyridoxal-5′-phosphate (vitamin B6) menaquinone (vitamin K) folic acid
*Carotenoids and flavonoids*	α and β-carotene β-cryptoxanthin lutein lycopene zeaxanthin
*Fatty acids*	conjugated linoleic acid 9,11–18: ct2 (CLA) n-3 long-chain polyunsaturated fatty acids (LCPUFA)
*Thiol containing* *amino acids*, *peptides* *and proteins*	cysteine methionine glutathione thioredoxin
*Binding and transport proteins*	α-lactalbumin ceruloplasmin lactoferrin transferrin osteopontin
*Other molecules*	bilirubin uric acid lysozyme immunoglobulins
*Growth factors*	erythropoietin
*Hormones*	melatonin leptin adiponectin

**Table 3:** Distribution of selected trace elements in the three fractions of human milk according to [20,26,37].

% of Total	Milk Fat/MFGM	Low-Molecule Aqueous Fraction/Whey (Protein Bound)	Casein/Micelle
*Selenium*	5–10% on the outer MFGM; increased in hindmilk	60–70%	20–35%
*Copper*	5–15%	75–80%; most bound to ceruloplasmin or albumin, some to citrate and free amino acids	rest
*Zinc*	29% bound to alkaline phosphatase embedded in the MFGM	28% bound to serum albumin 29 bound to citrate	14% predominantly bound to phosphoserine residues
*Manganese*	rest	70% and bound to lactoferrin	11%
*Iron*	33% MFGM, bound to xanthine oxidase	~30% in aqueous fraction 20–30% in whey fraction	10%

**Table 4:** Selected environmental and dietary contaminants in human milk. A summary of contaminants, identified by their Chemical Abstracts Service (CAS) registry number, found in human milk from studies performed in different countries. Chemical lipophilicity is expressed as LogP values ^a^. Contaminant-related definitions are summarized in Appendix B, Box A2.

Chemical Name	CAS No.	LogP ^a^	Source
polychlorinated biphenyls (PCBs) congeners			
PCB 118	31508-00-6	6.91	[120]
PCB 138	35065-28-2	7.51	[120,121]
PCB 153	35065-27-1	7.51	[120,121]
PCB 180	35065-29-3	8.11	[120,121]
polybrominated diphenyl ethers (PBDEs) congeners			
PBDE 47	5436-43-1	6.78	[120,122]
PBDE 99	60348-60-9	7.52	[120,122]
PBDE 100	189084-64-8	7.52	[120]
PBDE 153	68631-49-2	8.23	[120,122]
PBDE 154	207122-15-4	8.23	[120]
organochlorine-based plant protection products (PPPs)			
β-hexachlorocyclohexane (β-HCH, lindane, γ-HCH isomer and byproduct)	58-89-9	3.73	[123,124]
dimethyl tetrachloroterephthalate (dacthal, organochloride herbicide)	1861-32-1	4.03	[121]
hexachlorobenzene (HCB)	118-74-1	5.72	[121]
o,p′-DDE	3424-82-6	6.00	[120,121,124]
p,p′-DDE	72-55-9	6.05	[120,121,124]
o,p′-DDT	789-02-6	6.66	[120,121,124]
p,p′-DDT	50-29-3	6.71	[120,121,124]
methoxychlor	72-43-5	5.46	[124]
dieldrin	60-57-1	5.10	[124]
endosulfan	115-29-7	4.29	[124]
aldrin	309-00-2	5.80	[124]
heptachlor	76-44-8	5.52	[124]
polycyclic aromatic hydrocarbons (PAHs)			
benzo(a)pyrene	50-32-8	6.01	[125]
anthracene	120-12-7	4.30	[125]
pyrene	129-00-0	4.88	[125]
phenanthrene	85-01-8	4.30	[125]
indeno(1,2,3-cd) pyrene	193-39-5	6.54	[125]
fluorene	86-73-7	3.83	[125]
benzo(k)fluoranthene	207-08-9	5.99	[125]
benzo(ghi)perylene	191-24-2	6.56	[125]
perfluoralkylated substances (PFASs)			
perfluorooctane sulfonate (PFOS)	45298-90-6	1.74	[126,127,128,129]
perfluorooctanoid acid (PFOA)	335-67-1	4.33	[126,127,128,129]
perfluorononanoic acid (PFNA)	375-95-1	4.97	[127,128,129]
perfluoroalchilhexane sulfonic acid (PFHxS)	355-46-4	1.51	[127,128,129]
other PPPs			
chlorpyrifos/CPF (organophosphate)	2921-88-2	5.16	[120,123,130]
malathion (organophosphate)	121-75-5	2.32	[121,123]
permethrin/PERM (pyrethroid)	52645-53-1 (*cis*: 52341-33-0 *trans*: 52341-32-9)	6.61	[120,121]
propoxur (carbamate)	114-26-1	1.67	[121]
plasticizers (i.e., phthalates and bisphenols) and monoester metabolites			
diethylphthalate/DEP	84-66-2	2.31	N.D. ^#^
monoethylphthalate/MEP (diethylphthalate/DEP metabolite)	2306-33-4	1.67	[120]
di-(2-ethylhexyl) phthalate/DEHP	117-81-7	7.94	N.D. ^#^
mono-2-ethylhexyl phthalate/MEHP (di-(2-ethylhexyl) phthalate/DEHP metabolite)	4376-20-9	4.49	[120]
mono(2-ethyl-5-hydroxyhexyl) phthalate/MEHHP (DEHP metabolite)	40321-99-1	2.81	[120]
mono-(2-ethyl-5-oxohexyl) phthalate/MEOHP (DEHP metabolite)	40321-98-0	2.63	[120]
benzyl butyl phthalate/BBP	85-68-7	4.59	N.D. ^#^
monobenzyl phthalate/BzBP (benzyl butyl phthalate/BBP metabolite)	2528-16-7	2.89	[120]
di-*n*-butyl phthalate/DBP	84-74-2	4.43	N.D. ^#^
mono-*iso*-butyl phthalate/miBP (di-*n*-butylphthalate/DBP and benzyl butyl phthalate/BBP metabolite)	30833-53-5	2.42	[120]
mono-*n*-butyl phthalate/MnBP (DBP and BBP metabolite)	34-74-2	2.73	[120]
bisphenol A/BPA	80-05-7	3.37	[120]
TBBPA	79-94-7	6.83	[131]
mycotoxins and metabolites			
aflatoxin M1 (AFM1)	6795-23-9	0.90	[132,133,134,135]
aflatoxin B1 (AFB1)	1162-65-8	1.48	[133,134]
ochratoxin A (OTA)	303-47-9	1.74	[132,133,134,135]
deoxynivalenol (DON)	51481-10-8	−0.97	[133,134]
fumonisin B2 (FB2)	116355-84-1	2.66	[133]
zearalenone (ZEA)	17924-92-4	3.41	[132,133,135]
T-2 toxin (T2)	21259-20-1	2.46	[133]
toxic trace elements			
arsenic	7440-38-2	−0.61	[136,137]
cadmium	7440-43-9	−1.11	[138]
mercury	7439-97-6	−0.26	[138]
lead	7439-92-1	2.93	[138]

^a^ LogP: the octanol-water partition coefficient (LogP) has been calculated using the freely accessible method developed at Molinspiration [139]; LogP is reported here to provide basic information about the probability of each contaminant to enter milk depending on their lipophilicity (higher positive LogP values). ^#^ The plasticizers’ parental compounds are listed here to highlight the LogP difference with their metabolites, not for their presence in human milk [120]. The transition into milk does not have to be immediate: Activation of maternal body stores during lactation is a delayed route of transition: After maternal intake lipophilic compounds rapidly accumulate in white adipose tissue (WAT) and form a long-lasting depot there [140]. Using WAT biopsies in epidemiological studies as integrating markers of exposure to contaminants over long periods has been proposed as consequence [141].

## Abbreviations

•OH	hydroxyl radical
AAP	American Academy of Pediatrics
ABCG2	ATP-binding cassette sub-family G member 2
ADHD	attention-deficit/hyperactivity disorder
AFB1	aflatoxin B1
AFM1	aflatoxin M1
AhR	aryl hydrocarbon receptor
AKT1	stock A strain k thymoma/transforming protein kinase 1
AR	androgen receptor
ATP	adenosine triphosphate
BaP	benzo(a)pyrene
BBP	benzyl butyl phthalate
BPA	bisphenol A
BzBP	monobenzyl phthalate
CAS no.	Chemical Abstracts Services registry number
CAT	catalase
CCAAT	cytosine-cytosine-adenosine-adenosine-thymidine box motif
CDC	Center of Disease Control and Prevention
CLA	conjugated linoleic acid
CPF	chlorpyrifos
CpG	5′-C-phosphate-G-3′
Dacthal	dimethyl tetrachloroterephthalate
DBP	di-*n*-butylphthalate
DCPA	dimethyl tetrachloroterephthalate
DDE	dichlorodiphenyldichloroethylene
DDT	dichlorodiphenyltrichloroethane
DEHP	di-(2-ethylhexyl) phthalate
DEP	diethylphthalate
DHA	docosahexaenoic acid
DOHaD	Developmental Origins of Health and Disease
DON	deoxynivalenol
ECHA	European CHemicals Agency
ED	endocrine disruptor(s)
EFSA	European Food Safety Authority
ER	estrogen receptor
ERR	estrogen-related receptor
ESPGHAN	European Society for Paediatric Gastroenterology, Hepatology and Nutrition
FB2	fumonisin B2
Fe^2+^	ferrous iron
Fe^3+^	ferric iron
GR	glutathione reductase
GSH	glutathione
GSHPx	glutathione peroxidases
GST	glutathione S-transferase
H_2_O_2_	hydrogen peroxide
HCB	hexachlorobenzene
HIF	hypoxia-inducible factor
HOO•	hydroperoxyl radical
IGF2	insulin-like growth factor 2
IR	interquartile range
LCPUFA	long-chain polyunsaturated fatty acid(s)
LogP	octanol-water partition coefficient
MEHHP	mono(2-ethyl-5-hydroxyhexyl) phthalate
MEHP	mono-2-ethylhexylphthalate
MEOHP	mono-(2-ethyl-5-oxohexyl) phthalate
MEP	monoethylphthalate
MFG	milk fat globule
MFGM	milk fat globule membrane
miBP	mono-*iso*-butyl phthalate
MAPK	mitogen-activated protein kinase
MnBP	mono-*n*-butyl phthalate
NFκB	Nuclear factor kappa B
NICU	Neonatal Intensive Care Unit
non-POP	non-persistent organic pollutants
NOS	nitric oxide synthase
Nrf2	Nuclear factor erythroid 2-related factor 2
O_2_•^−^	superoxide anion radical
OTA	ochratoxin A
PAH	polycyclic aromatic hydrocarbons
PBDE	polybrominated diphenyl ethers congeners
PCB	polychlorinated biphenyls
PERM	permethrin
PFAS	per- and polyfluoroalkyl substance
PFHxS	perfluoroalchilhexane sulfonic acid
PFNA	perfluorononanoic acid
PFOA	perfluorooctanoid acid
PFOS	perfluorooctane sulfonate
Pg	pictogram
PI3K	Phosphoinositide 3-kinase
pKa	acid dissociation constant
POP	persistent organic pollutant
PPAR α,γ	peroxisome proliferator-activated receptor alpha, gamma
PPP	plant protection products
PVC	polyvinyl chloride
rER	rough Endoplasmic reticulum
RNS	reactive nitrogen species
ROO•	peroxyl radical
RON	reactive nitrogen species
RONS	reactive oxygen and nitrogen species
ROS	reactive oxygen species
SD	standard deviation
SEM	standard error of the mean
SOD	superoxide dismutase
T2	T-2 toxin
TBBPA	tetra bromo bisphenol A
TDS	testicular disgenesis syndrome
TR	thyroid receptor
VDR	vitamin D receptor
VLDL	very low density lipoprotein.
WAT	white adipose tissue
WHO	World Health Organization
XDH	xanthine oxidase/hydrogenase
ZEA	zearalenone
β-HCH	β-hexachlorocyclohexane

## Appendix A

Box A1 Milk-related terminology. **Lactogenesis** is the secretion of large amounts of milk after having given birth. In women, lactogenesis starts about 2–3 days postpartum [83], which explains the three milk stages and their timing. Timing and duration of milk or **lactation stages** are not clearly defined. Generally, **colostrum** is a pre-milk and the earliest milk-like secretion expressed after birth and in the first week postpartum (day 0–5). **Transitional milk** most often describes the following week or two, i.e., days 6–14/15 or 21. Milk is considered **mature** at latest four weeks postpartum yet some studies use day 16 to indicate that milk production has stabilized [23,56]. **Foremilk** is the watery protein and lactose rich first milk released from the mammary gland at the start of a breastfeeding (or milking) session. **Hindmilk** is the late milk from the same breast in the same breastfeeding session. Hindmilk is about twice as rich in fat as foremilk and contains the fat-soluble vitamins [9,83,243].

## Appendix B

Box A2 Contaminant-related terminology. **Xenobiotics** are synthetic chemicals released in the environment by industrial processes and/or by consumers’ products that can be found in any living organism, including humans. **Environmental and dietary contaminants** are those xenobiotics found in living organisms, including humans, whose exposure in the general population is mainly occurring via contamination of the food chain and dietary intake of foods and water. **Endocrine disruptors** (EDs) are those xenobiotics and/or dietary and environmental contaminants able to interfere with the mammalian endocrine system causing an adverse health effect. A formal definition of ED being the following one “an exogenous substance or mixture that alters function(s) of the endocrine system and consequently causes adverse health effects in an intact organism, or its progeny, or (sub)populations” [105]. **Epigenetic regulators** are those EDs affecting gene expression without altering nuclear DNA sequences and potentially acting with transgenerational effects on the endocrine-regulated pathways.
